# Sequential treatment in advanced epidermal growth factor receptor-mutated lung adenocarcinoma patients receiving first-line bevacizumab combined with 1st/2nd-generation EGFR-tyrosine kinase inhibitors

**DOI:** 10.3389/fonc.2023.1249106

**Published:** 2023-10-03

**Authors:** Ping-Chih Hsu, Chun-Yao Huang, Yu-Ching Lin, Suey-Haur Lee, Li-Chung Chiu, Chiao-En Wu, Scott Chih-Hsi Kuo, Jia-Shiuan Ju, Allen Chung-Cheng Huang, Ho-Wen Ko, Chin-Chou Wang, Cheng-Ta Yang

**Affiliations:** ^1^ Division of Thoracic Medicine, Department of Internal Medicine, Chang Gung Memorial Hospital at Linkou, Taoyuan, Taiwan; ^2^ Department of Medicine, College of Medicine, Chang Gung University, Taoyuan, Taiwan; ^3^ Department of Pulmonary and Critical Care, Buddhist Tzu Chi General Hospital, New Taipei City, Taiwan; ^4^ Division of Thoracic Oncology, Department of Respiratory and Critical Care Medicine, Chang Gung Memorial Hospital, Chiayi County, Taiwan; ^5^ Department of Respiratory Care, Chang Gung University of Science and Technology, Chiayi County, Taiwan; ^6^ Division of Pulmonary & Critical Care Medicine, Kaohsiung Chang Gung Memorial Hospital, Kaohsiung, Taiwan; ^7^ Division of Hematology-Oncology, Department of Internal Medicine, Chang Gung Memorial Hospital at Linkou, Taoyuan, Taiwan; ^8^ Department of Internal Medicine, Taoyuan Chang Gung Memorial Hospital, Taoyuan, Taiwan; ^9^ Department of Respiratory Therapy, College of Medicine, Chang Gung University, Taoyuan, Taiwan

**Keywords:** epidermal growth factor receptor mutation, tyrosine kinase inhibitor, bevacizumab, lung adenocarcinoma, T790M, osimertinib

## Abstract

**Introduction:**

The clinical outcomes of sequential treatment of advanced epidermal growth factor receptor (EGFR)-mutated non-small cell lung cancer (NSCLC) patients with first-line bevacizumab combined with 1st/2nd-generation EGFR-TKIs are unclear. Thus, we aimed to analyze the outcomes of these patients.

**Methods:**

Between January 2015 and December 2020, data for 102 advanced EGFR-mutated lung adenocarcinoma patients receiving first-line bevacizumab combined with erlotinib or afatinib followed by treatments at multiple institutions were retrospectively analyzed. All patients with progressive disease (PD) after first-line therapy underwent secondary T790M mutation detection.

**Results:**

The secondary T790M mutation positive rate of all study patients was 57.9%. First-line erlotinib use and progression-free survival (PFS) after first-line therapy > 12 months were positively associated with the T790M mutation (P <0.05). The response rates (RRs) to second-line treatments were 51.7% and 22.7% for the osimertinib and nonosimertinib groups, respectively (P = 0.001). The median PFS associated with second-line osimertinib and nonosimertinib therapy was 13.7 and 7.1 months, respectively (hazard ratio (HR) = 0.38; 95% confidence interval (CI), 0.23–0.63; P< 0.001). Patients with a secondary T790M mutation receiving second-line osimertinib treatment had a median overall survival (OS) of 54.3 months, and the median OS was 31.9 months for non-T790M-mutated patients receiving second-line nonosimertinib treatments (HR = 0.36; CI: 0.21–0.62, P < 0.001).

**Conclusion:**

The majority of acquired resistance to first-line bevacizumab combined with 1st/2nd-generation EGFR-TKIs is associated with the T790M mutation. Sequential osimertinib treatment in patients with positive secondary T790M mutation is associated with better outcomes among these patients.

## Graphical Abstract

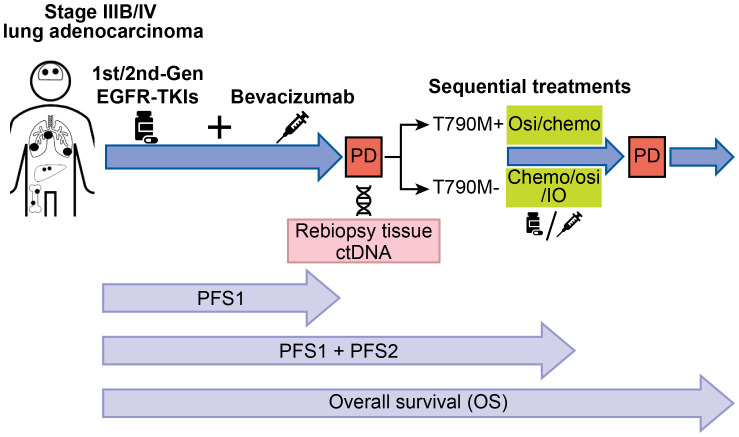

## 1 Introduction

The epidermal growth factor receptor (EGFR) and its downstream signaling pathway play crucial roles in the tumorigenesis of human non-small cell lung cancer (NSCLC) (1, 2). EGFR mutations account for the majority of oncogenic driver mutations in East Asian lung adenocarcinoma patients, and the incidence rate ranges from 40 to 55% (3, 4). The exon 19 deletion (in-frame deletions within exon 19) and L858R (a point mutation at codon 858 within exon 21 by leucine-to-arginine substitution) are the two most frequent (approximately 90%) EGFR mutations in lung adenocarcinoma (1–4). First- and second-generation EGFR tyrosine kinase inhibitors (TKIs), such as erlotinib and afatinib, have been demonstrated to be effective for treating advanced NSCLC harboring exon 19 deletion or L858R EGFR mutations (60-80% objective response rate (RR) and 10-14 months progression-free survival (PFS)) in several prospective clinical trials (5–7). Therefore, erlotinib and afatinib have been used as standard first-line therapies for advanced NSCLC harboring EGFR mutations worldwide.

The vascular endothelial growth factor receptor (VEGFR) signaling pathway has been reported to be involved in tumor growth and progression in various cancer cells (8, 9). Vascular endothelial growth factor (VEGF) is the ligand of VEGFR, and a previous study showed that EGFR-mutated NSCLC cells had increased VEGF protein expression levels compared with wild-type EGFR NSCLC cells (10). Another previous study showed that increased VEGF mRNA expression in plasma and tumor stroma was associated with resistance to EGFR-TKIs, and combined EGFR-TKIs and VEGF inhibitors had synergistic antitumor effects in an NSCLC mouse model (11). Bevacizumab is a humanized monoclonal antibody targeting VEGF and is used as an angiogenesis agent in anticancer therapies (8, 12). The efficacy of bevacizumab in combination with erlotinib or afatinib for the treatment of untreated advanced EGFR-mutated lung adenocarcinoma has been explored in several previous pivotal clinical trials and clinical studies (13–17). In these previous studies, the combination of erlotinib or afatinib with bevacizumab was demonstrated to have an objective RR of 80% and PFS of 13~24 months (13–17). Therefore, combination therapies have been suggested as a first-line therapeutic option for advanced lung adenocarcinoma patients with EGFR mutations.

The secondary T790M EGFR mutation is the most frequent cause of acquired resistance to first- and second-generation EGFR-TKIs (40%~60%) (18, 19). Osimertinib is a third-generation EGFR-TKI with active targeting of the T790M mutation and was shown to have promising efficacy (71% RR and 10.1 months PFS) in a pivotal clinical trial (AURA3 trial) (20). Therefore, osimertinib has been approved as a therapy for advanced NSCLC patients with secondary T790M mutation with progressive disease (PD) after first- or second-generation EGFR-TKI therapies.

The secondary T790M EGFR mutation appears in advanced lung adenocarcinoma with acquired resistance due to prior bevacizumab treatment combined with erlotinib or afatinib, and osimertinib is administered as a subsequent therapy for T790M-positive patients (15, 16). However, the clinical factors associated with the appearance of a positive T790M mutation in patients receiving first-line combination therapy remain unclear. Thus, we sought to analyze the survival outcomes of patients receiving first-line bevacizumab combined with erlotinib or afatinib followed by sequential systemic therapies (e.g., osimertinib or chemotherapy) after acquired resistance to first-line combination therapy.

## 2 Materials and methods

### 2.1 Patients and EGFR mutations

**Figure 1:**
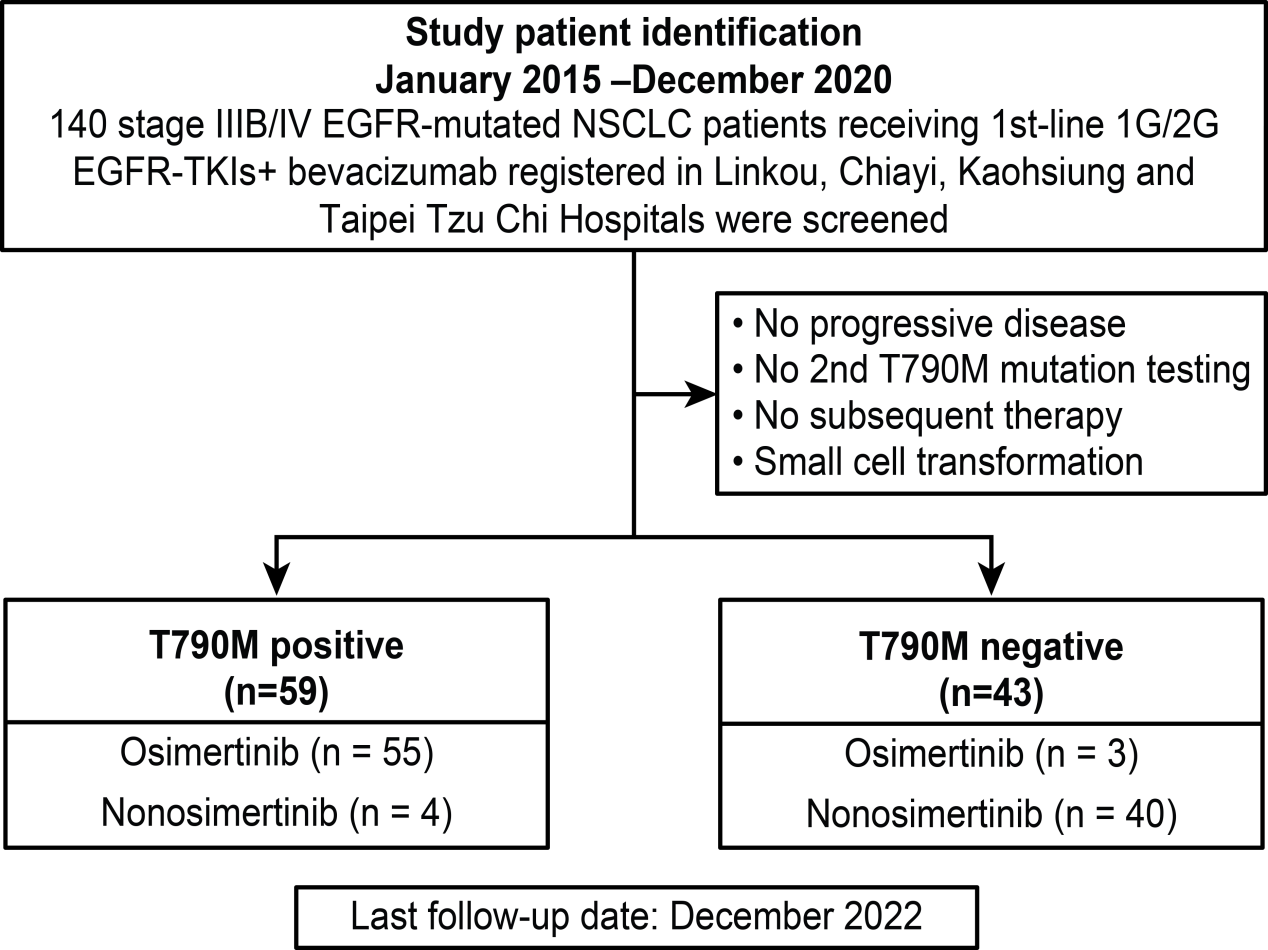
Flow chart of the included study patients.

Data from all study patients were retrospectively retrieved from the cancer center database of Linkou, Kaohsiung, Chiayi Chang-Gung Memorial hospitals (CGMHs) and Taipei Tzu Chi Hospital. Between January 2015 and December 2020, 140 advanced lung adenocarcinoma patients with EGFR mutations receiving bevacizumab combined with first- or second-generation EGFR-TKIs as first-line therapy were screened. The inclusion criteria were as follows: (1) patients with primary EGFR mutations without *de novo* T790M; (2) patients with PD after first-line therapy; (3) patients with secondary EGFR T790M mutation tests; and (4) patients receiving subsequent systemic therapies. The exclusion criteria were as follows: (1) no PD after first-line therapy; (2) no tests to detect secondary EGFR T790M mutation; (3) no subsequent systemic therapy administered; and (4) small cell transformation. The summary of study subject screening is summarized in **Figure 1**.

Amplified refractory mutation system–Scorpion (ARMS/S) assays or next-generation sequencing (NGS) was used to detect primary EGFR mutations and secondary T790M mutations in patients with PD after first-line therapy. The NGS panel used in this study was the same as that described in a previous study (16).

### 2.2 Treatment response, survival evaluation, and follow-up

The baseline stages at initial diagnosis of all subjects were determined by computed tomography (CT) with contrast medium enhancement, fluorodeoxyglucose (FDG)-positron emission tomography (PET), and magnetic resonance imaging (MRI) of the brain. All study patients underwent whole-body CT scans every 3 to 4 months to evaluate treatment responses. Additional imaging studies such as sonogram, plain films, MRI and FDG-PET were ordered by physicians based on their need for assistance in disease status assessment.

Response Evaluation Criteria in Solid Tumors (RECIST) version 1.1. was used to assess treatment responses. The treatment responses were classified as complete response (CR), partial response (PR), stable disease (SD), and PD. The length of PFS was defined from the treatment start date to the date of first PD images detected or last follow-up. The length of overall survival (OS) was defined from the starting date of first-line therapy to the date of mortality recorded. For patients who survived through the time point of last follow-up (December 31, 2022), the OS was censored at the last recorded clinical visit date.

### 2.3 Statistical analysis

Categorical variables of study subjects are presented as quantitative variables, and age is presented as the mean ± standard deviation (SD). Cox regression with univariate and multivariate analyses was used to determine the clinical factors associated with the T790M mutation rates. PFS and OS were estimated by Kaplan–Meier survival curves, and two-sided P values were considered statistically significant when they were smaller than 0.05. IBM SPSS Statistics version 22.0 (SPSS Corp., Chicago, IL, USA) was used to perform univariate and multivariate Cox regression analyses. The PFS and OS survival curves were generated by using GraphPad Prism (Version 5.0; GraphPad Software, San Diego, CA, USA).

## 3 Results

### 3.1 Baseline patient clinical characteristics and information on sequential treatments

All baseline clinical characteristics of the 102 study patients are shown in **Table 1**. Ninety-nine (97.1%) patients underwent tissue rebiopsy, and 8 (7.8%) patients had plasma circulating tumor (ct)-DNA liquid biopsy for secondary T790M mutation tests. Five (4.9%) patients had both tissue rebiopsy and ctDNA tests, and 3 (2.9%) patients had ctDNA tests only. Among the 5 (4.9%) patients with both tissue rebiopsy and ctDNA assessment, all the rebiopsy tissues were tested by using NGS, and according to the NGS results, 4 (3.9%) patients were negative for the T790M mutation, and the other 1 (1%) was positive. All five (4.9%) patients were negative for the T790M mutation based on ctDNA tests. In the 3 (2.9%) patients who underwent ctDNA testing alone, 2 (1.9%) were positive for the T790M mutation, and 1 (1%) was negative. In the 59 (57.9%) patients positive for the T790M mutation, 55 (53.9%) were administered osimertinib, 3 (2.9%) received platinum-based doublet chemotherapy, and 1 (1%) was switched from first-line erlotinib to afatinib and continued to receive bevacizumab as 2nd-line therapy. In 43 (42.1%) patients negative for the T790M mutation, 3 (2.9%) received osimertinib, 39 received chemotherapy-based therapy, and 1 patient received single pembrolizumab (anti-programmed death-1 (PD-1) inhibitor) as 2nd-line therapy. Twenty-three (22.5%) patients received antiangiogenic agents, including bevacizumab and ramucirumab (anti-VEGFR inhibitor), as second-line therapy. Four (3.9%) patients received second-line osimertinib with continuation of bevacizumab, and 6 (5.9%) patients received ramucirumab combined with osimertinib as second-line therapy. Ten (9.8%) patients received bevacizumab combined with chemotherapy, and 4 (3.9%) of the 10 (9.8%) patients received bevacizumab combined with chemotherapy and atezolizumab (anti-programmed cell death-ligand 1 (PD-L1) inhibitor). Three (2.9%) patients received chemotherapy combined with ramucirumab.

**Table 1:** Baseline characteristics of all study patients.

Total	N = 102 (%)
Sex	
Male	37 (36.3%)
Female	65 (63.7%)
Age, years (mean ± SD)	57.71 ± 11.02
ECOG PS	
0-1	84 (82.4%)
≧2	18 (17.6%)
Smoking status	
Nonsmoker	25 (24.5%)
Former/current smoker	77 (75.5%)
Histology	
Adenocarcinoma	102 (100%)
Stage	
IIIB	4 (3.9%)
IV	98 (96.1%)
EGFR mutation	
L858R	48 (47.1%)
Exon 19 deletion	52 (51.0%)
Others*	2 (1.9%)
Metastatic sites	
Pleural effusion	31 (30.4%)
Brain	33 (32.4%)
Bone	43 (42.2%)
Liver	18 (17.6%)
Adrenal	6 (5.9%)
First-line EGFR-TKIs + bevacizumab	
Erlotinib	53 (52.0%)
Afatinib	49 (48.0%)
Secondary EGFR-T790M mutation detection methods	
Tissue rebiopsy	99 (97.1%)
Plasma circulating tumor(ct)-DNA	8 (7.8%)
Secondary T790M mutation	
Positive	59 (57.9%)
Negative	43 (42.1%)
Subsequent treatments	
Osimertinib	58 (56.9%)
Chemotherapy	42 (41.1%)
Other EGFR-TKI**	1 (1%)
Anti-PD-1/PD-L1 immune checkpoint inhibitors (ICIs)	9 (8.8%)
Chemotherapy + ICI	8 (7.8%)
ICI alone	1 (1%)
Anti-angiogenesis agents	
Bevacizumab	14
Ramucirumab	9

SD, standard deviation; ECOG PS, Eastern Cooperative Oncology Group performance status; EGFR, epidermal growth factor receptor; TKI, tyrosine kinase inhibitor; PD-1, programmed death-1; PD-L1, programmed cell death-ligand 1.

* 1 G719X, 1 S768I.

**Afatinib.

### 3.2 Clinical factors associated with secondary EGFR T790M mutation after first-line bevacizumab combined with 1st-/2nd-generation EGFR-TKIs

The clinical factors associated with secondary T790M mutation after first-line therapy in this study were analyzed by using univariate and multivariate Cox regression (**Table 2**). In univariate analysis, the primary exon 19 deletion mutation had a trend of a higher secondary T790M mutation-positive rate than the primary L858R mutation, but no statistical significance was achieved. First-line bevacizumab combined with erlotinib had a significantly higher secondary T790M mutation-positive rate than bevacizumab combined with afatinib. In addition, a longer PFS (> 12 months) while on first-line treatment had a significantly higher T790M mutation rate than a shorter PFS (≦12 months). The multivariate analysis showed that first-line erlotinib use (vs. afatinib, odds ratio: 2.734, 95% confidence interval (CI): 1.144–6.531, P = 0.029) and longer PFS while on first-line therapy (vs. ≤12 months PFS, odds ratio: 2.958, 95% CI: 1.142–7.661, P = 0.025) were independent predictive factors associated with secondary T790M mutation. The clinical information comparison between patients treated with first-line afatinib plus bevacizumab and erlotinib plus erlotinib is shown in **Supplementary Table S1**.

**Table 2:** Univariate and multivariate analyses of predictors associated with acquired T790M mutation (n=59).

	Number of patients	T790M+ (%)	*P* value	Multivariate analysis	
				Odds ratio (95% CI)	*P* value
Basic data					
Sex			0.584		
Male	20	33.9 (%)			
Female	39	66.1 (%)			
Age (years)			0.466		
≦60	30	50.8 (%)			
> 60	29	49.2 (%)			
ECOG PS			0.219		
0-1	50	84.7 (%)			
≥2	9	15.3 (%)			
Smoking			0.830		
Nonsmoker	45	76.3 (%)			
Current/former	14	23.7 (%)			
EGFR mutation			0.087		
L858R	23	39.0 (%)			
Exon 19 deletion	35	59.3 (%)			
Others	1	1.7 (%)			
Stage			0.999		
IIIB	3	5.1 (%)			
IV	56	94.9(%)			
First-line TKI used					
Afatinib	23	39.0 (%)	0.033	1	
Erlotinib	36	61.0 (%)		2.734 (1.144-6.531)	0.029
PFS (months)			0.013		
≦12	10	16.9 (%)		1	
>12	49	83.1 (%)		2.958 (1.142-7.661)	0.025

ECOG PS, Eastern Cooperative Oncology Group performance status; CI, confidence interval; PFS: progression-free survival; EGFR, epidermal growth factor receptor; TKI, tyrosine kinase inhibitor.

### 3.3 Analysis of PFS and OS between the two first-line therapy groups

**Figure 2:**
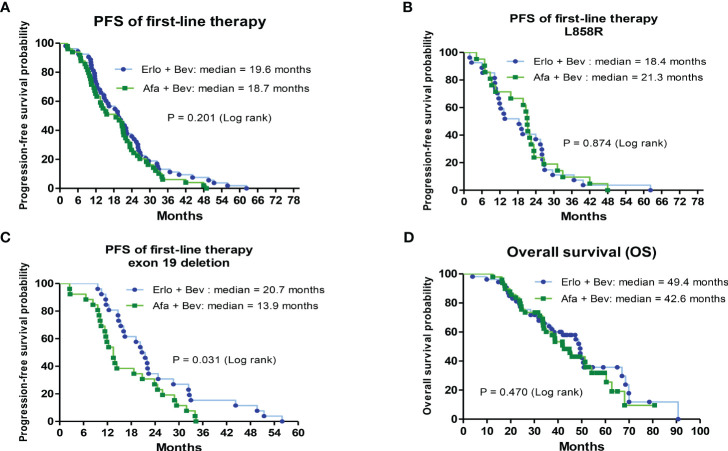
Analysis of progression-free survival (PFS) for first-line treatments and overall survival (OS) for first-line treatments by Kaplan–Meier survival curves. **(A)** Comparison of median PFS between bevacizumab combined with erlotinib or afatinib (HR = 1.05; 95% CI, 0.52–1.15; P= 0.201). **(B)** The median PFS between bevacizumab combined with erlotinib or afatinib in primary L858R-mutated patients (HR = 1.05; 95% CI, 0.59–1.87; P= 0.874). **(C)** The median PFS between bevacizumab combined with erlotinib or afatinib in primary exon 19 deletion-mutated patients (HR = 0.53; 95% CI, 0.29–0.94; P= 0). **(D)** The median OS between bevacizumab combined with erlotinib or afatinib (HR = 0.841; 95% CI, 0.51–1.38; P= 0.470).

The PFS and OS of the 2 first-line therapies were analyzed by Kaplan–Meier survival curves. There was no significant difference in the median PFS of first-line bevacizumab combined with erlotinib and first-line bevacizumab combined with afatinib (19.6 vs. 18.7 months, hazard ratio (HR) = 1.05; CI: 0.52–1.15, P = 0.201) (**Figure 2A**). Patients with different primary EGFR mutations (L858R and exon 19 deletion) were divided into 2 groups to analyze the PFS associated with first-line therapies. In L858R-mutated patients, the median PFS was 18.4 and 21.3 months for the bevacizumab combined with erlotinib group and bevacizumab combined with erlotinib group, respectively (HR = 1.05; CI: 0.59–1.87, P = 0.874) (**Figure 2B**). For patients with primary exon 19 deletion mutations, the median PFS of the first-line bevacizumab combined with erlotinib group was significantly higher than that of the first-line bevacizumab combined with afatinib group (20.7 vs. 13.9 months, HR = 0.53; CI: 0.29–0.94, P = 0.031) (**Figure 2C**). Regarding OS, no significant difference was noted between the 2 groups of patients receiving first-line bevacizumab combined with erlotinib and first-line bevacizumab combined with afatinib (median OS: 49.4 VS. 42.6 months, HR = 0.841; CI: 0.51–1.38, P = 0.470) (**Figure 2D**). Patients with baseline brain metastasis were analyzed, and the results are shown in **Supplementary Figure S1**. The treatment response rate of first-line combination therapy was 84.8%, and median PFS was 14.7months in patients with baseline brain metastasis (**Supplementary Figures S1A, B**). The median OS of patients with baseline brain metastasis was 34.3 months (**Supplementary Figure S1C**).

### 3.4 Treatment outcomes of patients with different T790M mutation statuses and subsequent treatments

Most secondary T790M mutation-positive patients (56 of 59 = 94.9%) who received osimertinib as second-line therapy were divided into osimertinib and nonosimertinib groups for comparison. Second-line osimertinib treatment had a significantly higher objective RR than nonosimertinib therapy (51.7% vs. 22.7%, P = 0.001) (**Table 3**). All 3 patients who underwent liquid biopsy alone received osimertinib as subsequent treatments, and all patients had SD to osimertinib therapy. The PFS of the 3 patients ranged from 6.37 to 22.17 months. The patient who was T790M negative in liquid biopsy had a 14.83 PFS on osimertinib therapy.

**Table 3:** Clinical response to 2^nd^-line therapy.

	Osimertinib N =58 (%)	Nonosimertinib N = 44 (%)	P value
CR	0	0	0.001
PR	30 (51.7%)	10 (22.7%)	
SD	26 (44.8)	24 (54.5%)	
PD	2 (3.5%)	10 (22.7%)	
RR (%)	51.7	22.7	
DCR (%)	96.5	77.3	

CR, complete response; PR, partial response; SD, stable disease; PD, progressive disease; RR, response rate; DCR, disease control rate.

**Figure 3:**
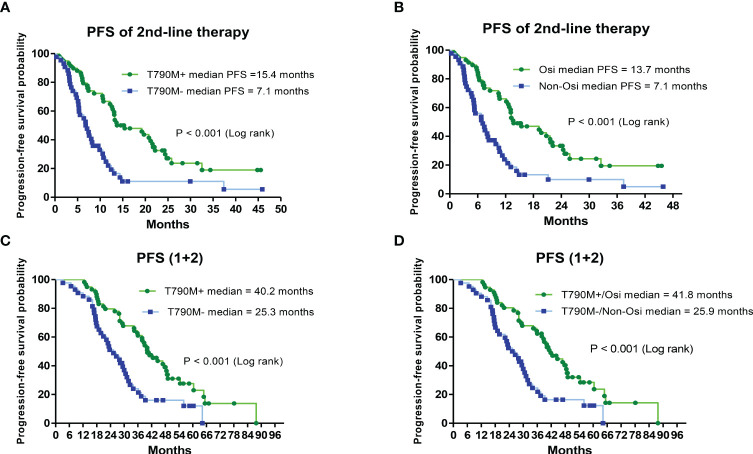
Analysis of progression-free survival (PFS) of second-line and first-line plus second-line (PFS1 + 2) therapies by Kaplan–Meier survival curves. **(A)** Comparison of PFS of second-line treatments between T790M mutation-positive and T790M mutation-negative patients (HR = 0.37; 95% CI, 0.22–0.61; P< 0.001). **(B)** Comparison of PFS between second-line osimertinib and nonosimertinib treatments (HR = 0.38; 95% CI, 0.23–0.63; P< 0.001). **(C)** Comparison of PFS (1 + 2) between T790M mutation-positive and T790M mutation-negative patients (HR = 0.39; 95% CI, 0.24–0.65; P< 0.001). **(D)** Comparison of PFS (1 + 2) between second-line osimertinib and nonosimertinib treatments (HR = 0.39; 95% CI, 0.23–0.65; P< 0.001).

Patients with secondary T790M mutation and second-line therapy had a significantly longer median PFS than those without T790M mutation (15.4 vs. 7.1 months, HR = 0.37; CI: 0.22–0.61, P < 0.001) (**Figure 3A**). The median PFS of those who received second-line osimertinib therapy was significantly longer than that of those who received nonosimertinib therapy (13.7 vs. 7.1 months, HR = 0.38; CI: 0.23–0.63, P < 0.001) (**Figure 3B**). The length of PFS of patients who received first-line plus second-line treatments (PFS1 + PFS2) was evaluated. Patients with a secondary T790M mutation had a significantly longer median PFS (1 + 2) than those without a T790M mutation (40.2 vs. 25.3 months, HR = 0.39; CI: 0.24–0.65, P < 0.001) (**Figure 3C**). Patients with a secondary T790M mutation who received 2nd-line osimertinib had a significantly longer median PFS (1 + 2) than those without a T790M mutation who received nonosimertinib therapy (41.8 vs. 25.9 months, HR = 0.39; CI: 0.23–0.65, P < 0.001) (**Figure 3D**).

**Figure 4:**
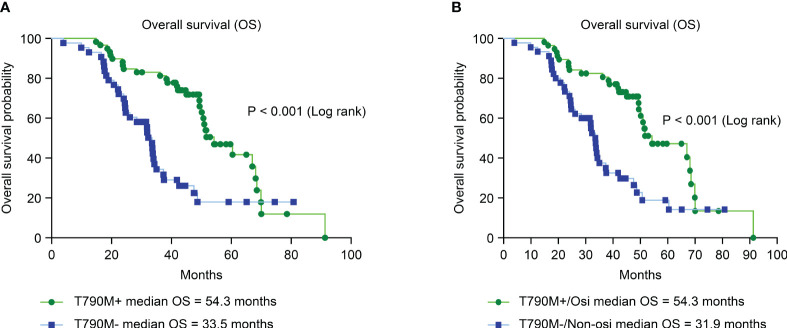
Analysis of overall survival (OS) between different T790M mutation statuses and second-line treatments by Kaplan–Meier survival curves. **(A)** Comparison of OS between T790M mutation-positive and T790M mutation-negative patients (HR = 0.34; 95% CI, 0.19–0.59; P< 0.001). **(B)** Comparison of OS between second-line osimertinib in T790M-positive and nonosimertinib in T790M-negative patients (HR = 0.36; 95% CI, 0.21–0.62; P< 0.001).

We further analyzed the OS of patients with different secondary EGFR T790M mutation statuses and subsequent treatments. Patients with a secondary T790M mutation had a significantly longer median OS than those without a T790M mutation (54.3 vs. 33.5 months, HR = 0.34; CI: 0.19–0.59, P < 0.001) (**Figure 4A**). Patients with secondary T790M mutation who received osimertinib as subsequent treatment had a significantly longer median OS than those without T790M mutation who received a nonosimertinib subsequent therapy (54.3 VS. 31.9 months, HR = 0.36; CI: 0.21–0.62, P < 0.001) (**Figure 4B**).

## 4 Discussion

The results of this study provide some important clinical information regarding sequential treatments for advanced EGFR-mutated NSCLC patients receiving first-line bevacizumab combined with 1st/2nd-generation EGFR-TKIs. First, the secondary T790M mutation rate after PD in this study was 57.9%. Second, the use of erlotinib in first-line therapy and PFS > 12 months were identified as independent predictive factors associated with higher secondary T790M mutation rates. Third, T790M-mutated patients receiving subsequent osimertinib had a significantly better treatment response and longer PFS than those without the T790M mutation receiving nonosimertinib therapy. In addition, T790M-mutated patients receiving subsequent osimertinib had significantly longer OS than those without the T790M mutation.

The acquired T790M mutation rate in this study was 57.9% and was consistent with that reported in previous studies (18, 21, 22). In contrast to previous studies, all the patients in this study received bevacizumab in addition to 1st/2nd-generation EGFR-TKIs, whereas most patients in previous studies received EGFR-TKI-alone therapies (18, 21, 22). The results of our study indicated that bevacizumab in addition to 1st/2nd-generation EGFR-TKIs does not alter the mechanism of acquired resistance in advanced primary EGFR-mutated NSCLC. Some previous studies showed that prior afatinib therapy was associated with a lower secondary T790M mutation-positive rate when compared with first-generation EGFR-TKIs, and these results were similar to those in our study (23, 24). Some previous studies showed that prior afatinib therapy was not associated with a lower secondary T790M mutation-positive rate and that this rate was even higher than that for first-generation EGFR-TKIs (25, 26). Although there are differences among our study and previous studies, the acquired T790M mutation rates after afatinib therapy in previous studies ranged from 30-50% (22–26). In addition, the small sample sizes in these studies may have led to different statistical significances among these studies. A long PFS of prior EGFR-TKI therapy (> 12 months) was identified as a predictive factor associated with acquired T790M mutation positive rates in previous studies, which was similar to the result in this study (22–26). A previous study showed that prolonging afatinib therapy in EGFR-mutated NSCLC by adding bevacizumab led to a positive acquired T790M mutation conversion, and the results in the same study suggested that prolonging afatinib therapy may induce the clonal selection of acquired T790M-mutated NSCLC cells (27). This clonal selection hypothesis may explain why long PFS of prior 1st/2nd-generation EGFR-TKI therapies is associated with an increased secondary T790M mutation rate. In the analysis of this study, bevacizumab combined with erlotinib had a significantly longer median PFS than bevacizumab combined with afatinib among patients with exon 19 deletion mutations. In addition, more patients in the bevacizumab combined with erlotinib treatment group had a longer PFS (> 12 months) than those in the bevacizumab combined with afatinib group (40 (75.5%) vs. 32 (65.3%)). Our study mainly focused on the rebiopsy results and subsequent therapies, and the study patients were retrospectively selected by selection criteria. Therefore, selection bias may lead to statistical significance in the median PFS between the first-line afatinib and erlotinib combined with bevacizumab groups in patients with exon 19 deletion mutations. Taken together, long treatment PFS is suggested to be the main factor associated with the occurrence of secondary T790M mutation, not afatinib and erlotinib therapies.

In the results of a previous clinical trial (AURA, NCT01802632), osimertinib had a 21% RR and a median PFS of 2.8 months for treating T790M mutation-negative patients with acquired resistance to prior EGFR-TKIs. The results of the AURA trial indicated that osimertinib is less effective in T790M-negative patients than in those with secondary T790M mutations after resistance to prior EGFR-TKI treatments (21). Before osimertinib was approved by the United States Food and Drug Administration (U.S. FDA, November 2015), platinum-based chemotherapy was the suggested subsequent treatment for patients who had PD after 1st/2nd-generation EGFR-TKI therapies (28, 29). Although osimertinib was approved for advanced NSCLC with acquired T790M mutation, chemotherapy has remained the clinically preferred subsequent treatment for T790M-negative patients with PD after 1st/2nd-generation EGFR-TKI therapies; furthermore, drugs targeting mutations other than T790M are still under investigation in clinical trials (29). Although immunotherapy, such as PD1/PD-L1 immune checkpoint inhibitors (ICIs), has been shown to improve the survival of advanced NSCLC patients without driver mutations (30), the survival benefit of immunotherapy is still very limited for advanced EGFR-mutated NSCLC patients (29, 30).

Osimertinib has been widely used as a late-line therapy for T790M-mutated NSCLC patients based on the results of AURA serial trials (20, 21, 31). In the survival analysis of the NEJ026 trial, patients treated with osimertinib in second-line or later-line therapies had significantly longer OS than those without osimertinib therapy after bevacizumab plus erlotinib or erlotinib alone treatment (32). A previous study also showed that T790M-mutated NSCLC patients receiving subsequent osimertinib therapy had significantly longer OS than those without acquired T790M mutation and subsequent osimertinib therapy. The same study showed that the use of 1st-generation or 2nd-generation EGFR-TKIs in first-line therapies did not affect OS (22). The results of our study are compatible with those shown in 2 previous clinical studies (22, 32). Taken together, these results indicated that the acquired T790M mutation is a key factor associated with OS in advanced EGFR-mutated NSCLC patients receiving 1st-generation or 2nd-generation EGFR-TKIs as first-line therapies.

Osimertinib is suggested as a first-line therapy for advanced EGFR-mutated NSCLC patients because a pivotal clinical trial (FLAURA) showed that osimertinib had a median PFS of 18.9 months and OS of 38.6 months, which were significantly longer than those of comparator therapies (10.2 months of median PFS and 31.8 months of median OS) (33). The median OS associated with first-line osimertinib in the FLAURA trial was 38.6 months (33). In the FLAURA trial (33), patients in the comparator arm received gefitinib or erlotinib alone treatments, whereas all patients in our study received bevacizumab in addition to erlotinib or afatinib.

A previous prospective trial (RELAY) demonstrated that erlotinib combined with ramucirumab had a significantly longer median PFS than erlotinib combined with placebo in untreated advanced EGFR-mutated NSCLC patients (19.4 vs. 12.4 months). Erlotinib combined with ramucirumab has been suggested as a first-line therapy choice for advanced EGFR-mutated NSCLC based on the results of the RELAY trial (34). However, patients with baseline brain metastasis were excluded by the RELAY trial, and the efficacy of ramucirumab combined with erlotinib in EGFR-mutated NSCLC patients with brain metastasis was not clear (34). In the NEJ026 study, 32% of study patients had baseline brain metastasis in the erlotinib combined with bevacizumab and erlotinib alone arms (14). A previous study also reported that bevacizumab in addition to EGFR-TKIs was more effective for brain metastasis control and prevention of the progression of brain metastasis than EGFR-TKI treatment alone in NSCLC with EGFR mutations (35). In addition, some previous studies reported that systemic administration of bevacizumab was effective for the control of NSCLC-related malignant pleural effusion (36). In this study, approximately 30% of patients had baseline brain metastasis and malignant pleural effusion. Regarding the concern regarding metastatic sites and study populations in previous studies, bevacizumab in combination with 1st-/2nd- EGFR-TKIs would be considered as first-line therapy for metastatic EGFR-mutated NSCLC patients.

In the NEJ026 clinical trial, patients who received osimertinib as second-line therapy had a median OS of approximately 50 months, and those who did not receive osimertinib treatments as second-line therapy had a median survival of approximately 40 months (32). In another retrospective clinical study (GioTag study), advanced EGFR-mutated NSCLC patients receiving first-line afatinib followed by osimertinib had a median OS of 37.6 months and 44.8 months in Asian patients (37, 38). A previous study also showed that patients receiving 1st/2nd-generation EGFR-TKIs followed by osimertinib had a median OS over 50 months (22). In this study, patients with acquired T790M mutations receiving subsequent osimertinib had a median OS of 54.3 months, which was compatible with the results of previous studies (22, 32, 37, 38). Together, these results suggest that the OS of advanced EGFR-mutated NSCLC patients who received bevacizumab in combination with 1st/2nd-generation EGFR-TKIs or afatinib alone followed by second-line osimertinib is not inferior to that of patients who received first-line osimertinib therapy. In addition, the median PFS of first-line bevacizumab combined with erlotinib or afatinib in advanced EGFR-mutated NSCLC patients shown by previous studies seems not inferior to first-line osimertinib (13–16). Therefore, bevacizumab combined with erlotinib or afatinib may be a choice of first-line therapy for advanced EGFR-mutated NSCLC patients other than osimertinib.

Rebiopsy, either liquid biopsy or direct tissue biopsy, for secondary T790M detection is recommended as standard care for advanced EGFR-mutated NSCLC with acquired resistance to 1st/2nd-generation EGFR-TKIs (21–26). Most patients in our study underwent tissue rebiopsy or liquid biopsy alone, and only a few (5 = 4.9%) patients underwent both procedures for tests. The second-line osimertinib therapy in this study had a 51.7% RR and a median PFS of 13.7 months, and these results indicated that the T790M mutation testing results were reliable in our study. Some previous studies have suggested that both liquid and tissue rebiopsy be performed for NGS tests because repeated biopsy by liquid or tissue increased the T790M detection rate and may also detect other genomic alterations for optimal subsequent treatment (39–41). In our study, all 5 patients who underwent both liquid and tissue rebiopsies were T790M-negative in liquid biopsy, and one was T790M-positive in tissue rebiopsy. This result indicated that a repeated tissue biopsy converts T790M-negative to T790M-positive results in some patients and is compatible with the findings of previous studies (39–41). In the 3 patients who underwent liquid biopsy alone, 2 had T790M-positive results, and 1 was T790M-negative. Although repeated rebiopsy has been recommended to increase the diagnostic accuracy and T790M positive rate, most patients received only one tissue rebiopsy. The main concerns regarding why patients did not receive repeated biopsies include personal acceptance, procedure-related adverse events, and tumor site procedure-unapproachable tumor sites such as tiny distant metastases (42, 43). Taken together, these findings explain why most patients have a low willingness to undergo repeated tissue rebiopsy in real-world clinical practice.

Small cell lung cancer transformation is a rare (<5%) acquired resistance to previous EGFR-TKI treatment (44). In this study, 2 patients had small cell transformation according to tissue rebiopsy and were excluded from further analysis. For advanced EGFR-mutated NSCLC patients who experienced small cell transformation after previous EGFR-TKI treatments, chemotherapy with platinum-based regimens combined with etoposide is recommended if the patient has acceptable performance status (44). For the 3 patients who underwent liquid biopsies only in this study, all of them were controlled by subsequent osimertinib treatments. Small cell transformation has also been reported as a resistance mechanism to prior osimertinib therapy in a previous study (44). According to the clinical treatment response to osimertinib in the 3 patients who underwent liquid biopsies only, the possibility of small cell transformation was very low, and the 3 patients were still included in this study.

Some limitations of this study should be clarified. First, the study population was East Asian, and whether the secondary T790M mutation rate and outcomes in other ethnic populations are similar to our results is unclear. A recent phase III clinical trial (BEVERLY) investigating the combination of bevacizumab with erlotinib for the treatment of advanced EGFR-mutated NSCLC recruited study patients mainly in European countries (45). In this trial, 24 (49%) patients in the bevacizumab combined with erlotinib arm were reported to receive osimertinib as second-line therapy, but information on the acquired T790M mutation and outcomes was not available (45). Second, the first-line EGFR-TKIs administered in this study were erlotinib and afatinib, and no patients in this study received gefitinib (1st-generation) or dacomitinib (2nd-generation) as first-line treatments. Future studies may be needed to analyze the clinical outcomes of advanced EGFR-mutated NSCLC patients receiving first-line bevacizumab combined with gefitinib or dacomitinib. Finally, the use of multiple genomic alteration detection methods, such as NGS, in NSCLC with acquired resistance to previous bevacizumab combined with erlotinib therapy increases in clinical practice, and resistant genomic alterations other than T790M, such as MET, HER2 or BRAF, can be detected by NGS (29). Targeted therapies for the abovementioned genomic alterations have been developed and explored in clinical trials (29), and patients who receive new targeted therapies may have improved outcomes in the future.

## 5 Conclusion

Our study clearly demonstrated the clinical perspective regarding sequential treatments with first-line bevacizumab combined with 1st/2nd-generation EGFR-TKIs in advanced lung adenocarcinoma patients harboring EGFR mutations. Secondary T790M mutation detection tests and optimal use of osimertinib may yield favorable survival outcomes.

## Data availability statement

The raw data supporting the conclusions of this article will be made available by the authors, without undue reservation.

## Ethics statement

This multicenter observational study was approved by the institutional review board (IRB) of the Chang Gung Medical Foundation (no. 202000137B0 and no. 202100379B0) and Taipei Buddhist Tzu Chi General Hospital (no. 09-X-002). Obtaining consent from study subjects was waived by the IRB because of the retrospective nature of this study. All patients in this study received standard cancer care and treatments following the protocol of Chang Gung Medical Foundation and Taipei Buddhist Tzu Chi General Hospital cancer centers. All treatment and evaluation procedures were performed in accordance with the Helsinki Declaration. No identifiable subjective information, such as personal ID or birthday, was presented in this manuscript.

## Author contributions

Conception and design: P-CH, C-YH, C-TY. Acquisition of data: P-CH, C-YH, YL, S-HL, L-CC, H-WK, C-CW. Analysis and interpretation of data: P-CH, C-YH, YL, S-HL, L-CC, C-EW, SK, J-SJ, AH, H-WK, C-CW. Writing of the manuscript: P-CH, and C-TY. Review and revision of the manuscript: L-CC, C-EW, SK, H-WK, C-TY. Administrative and funding support: P-CH, C-YH, C-TY. All authors contributed to the article and approved the submitted version.
