# Supplementary figures and images for: Sequential treatment in advanced epidermal growth factor receptor-mutated lung adenocarcinoma patients receiving first-line bevacizumab combined with 1st/2nd-generation EGFR-tyrosine kinase inhibitors
Source: Front Oncol. 2023 Oct 3;13:1249106. doi: 10.3389/fonc.2023.1249106 (PMC10579797; doi:10.3389/fonc.2023.1249106)

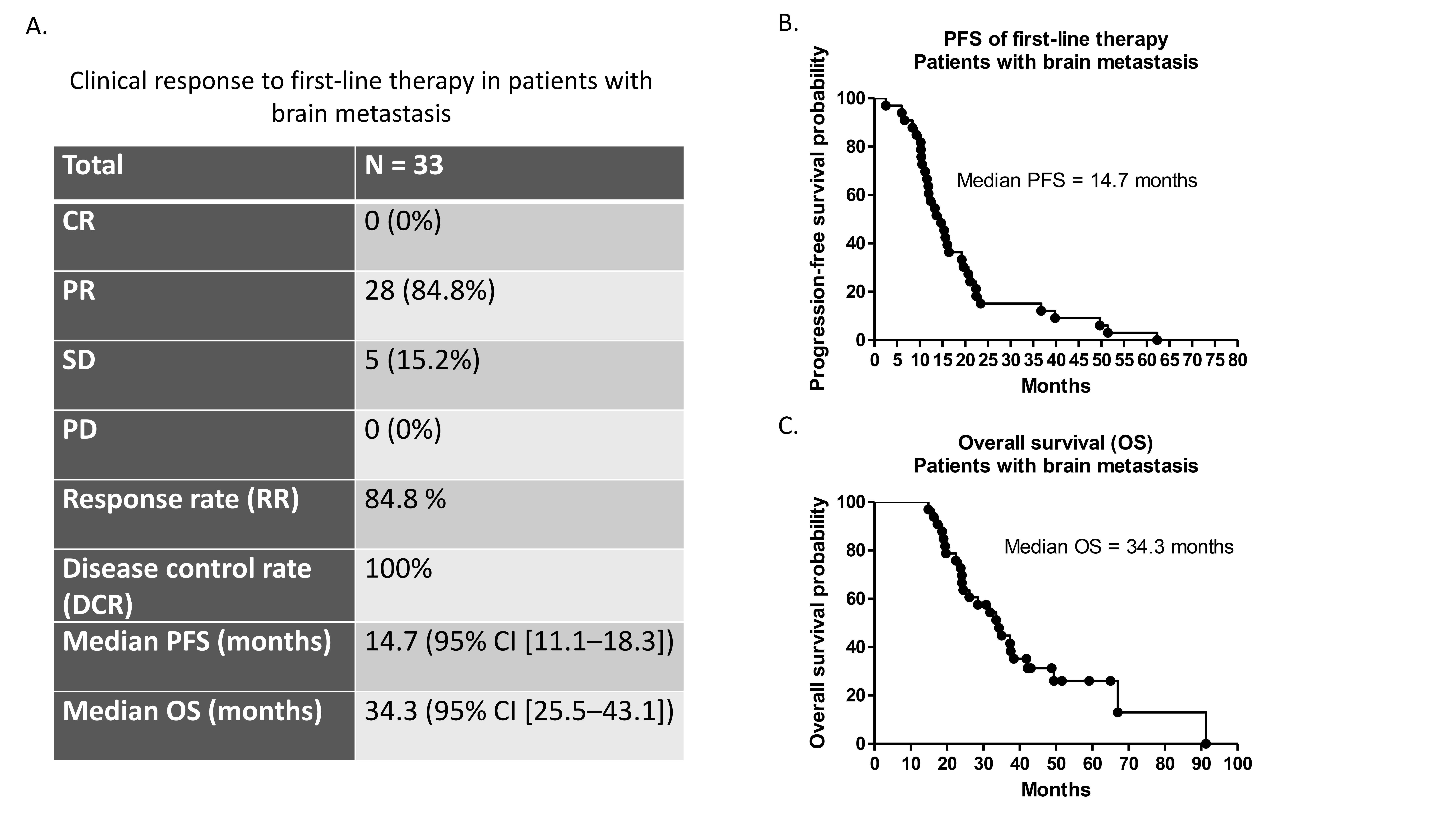

Supplement: Supplementary file 1 [file Image_1.tif]
