# Supplementary material for: Sequential treatment in advanced epidermal growth factor receptor-mutated lung adenocarcinoma patients receiving first-line bevacizumab combined with 1st/2nd-generation EGFR-tyrosine kinase inhibitors
Source: Front Oncol. 2023 Oct 3;13:1249106. doi: 10.3389/fonc.2023.1249106 (PMC10579797; doi:10.3389/fonc.2023.1249106)
Supplement: Supplementary file 2 [file Table_1.docx]

**Supplementary Table S1. Comparison of patient clinical characteristics between the first-line afatinib plus bevacizumab and erlotinib plus bevacizumab groups**

|  | Afa + Bev  N = 49 | Erl + Bev  N = 53 | P value |
| --- | --- | --- | --- |
| **Sex** |  |  | 0.359 |
| Male/female | 20/29 | 17/36 |  |
| **Age (mean ± SD)** | 56.4 ± 11.7 | 58.9 ± 10.3 | 0.307 |
| **ECOG PS** |  |  | 0.737 |
| 0-1 | 41 (83.7%) | 43 (81.1%) |  |
| 2 | 8 (16.2%) | 10 (18.9%) |  |
| **Smoking** |  |  | 0.996 |
| Former + Current | 12 (26.5%) | 13 (24.5%) |  |
| Nonsmoker | 37 (75.5%) | 40 (75.5%) |  |
| **Histology** |  |  |  |
| Adenocarcinoma | 49 (100%) | 53 (100%) |  |
| **Stage** |  |  | 0.271 |
| IIIB/IV | 3/46 | 1/52 |  |
| **EGFR mutations** |  |  | 0.273 |
| Exon 19 deletion | 26 (46.3%) | 26 (48.5%) |  |
| L858R | 21 (50.7) | 27 (51.5%) |  |
| Others* | 2 (3%) | 0 |  |
| **Secondary EGFR-T790M mutation**  **detection methods** |  |  | 1.000 |
| Tissue rebiopsy | 48 (98.0%) | 51 (96.2%) |  |
| Plasma circulating tumor(ct)-DNA | 4 (8.2%) | 4 (7.5%) |  |
| **Median PFS of first-line therapy**  **(months)** |  |  |  |
| All patients | 18.7 | 19.6 | 0.201 |
| Exon 19 deletion | 13.9 | 20.7 | 0.031 |
| L858R | 21.3 | 18.4 | 0.874 |
| **PFS (months)** |  |  |  |
| ≦12 | 17 (34.7%) | 13 (24.5%) | 0.284 |
| >12 | 32 (65.3%) | 40 (75.5%) |  |

Afa: afatinib; Bev: bevacizumab; Erl: erlotinib; SD: standard deviation; ECOG PS: Eastern Cooperative Oncology Group (ECOG) Performance; PFS: progression-free survival; *G719X and S768I
